# Feasibility and acceptability of mobile phone short message service as a support for patients receiving antiretroviral therapy in rural Uganda: a cross-sectional study

**DOI:** 10.7448/IAS.18.1.20311

**Published:** 2015-12-10

**Authors:** Jiho Kim, Wendy Zhang, Maureen Nyonyitono, Lillian Lourenco, Mastula Nanfuka, Stephen Okoboi, Josephine Birungi, Richard T Lester, Pontiano Kaleebu, Paula Munderi, David M Moore

**Affiliations:** 1 British Columbia Centre for Excellence in HIV/AIDS, Vancouver, Canada; 2 The AIDS Support Organization, Kampala, Uganda; 3 Faculty of Medicine, University of British Columbia, Vancouver, Canada; 4 Medical Research Council Joint Research Unit on AIDS, Uganda Virus Research Institute, Entebbe, Uganda

**Keywords:** health communication, ART reminders, ART adherence, mHealth

## Abstract

**Introduction:**

Mobile phone technologies have been promoted to improve adherence to antiretroviral therapy (ART). We studied the receptiveness of patients in a rural Ugandan setting to the use of short messaging service (SMS) communication for such purposes.

**Methods:**

We performed a cross-sectional analysis measuring mobile phone ownership and literacy amongst patients of The AIDS Support Organisation (TASO) in Jinja, Uganda. We performed bivariate and multivariate logistic regression analyses to examine associations between explanatory variables and a composite outcome of being literate and having a mobile phone.

**Results:**

From June 2012 to August 2013, we enrolled 895 participants, of whom 684 (76%) were female. The median age was 44 years. A total of 576 (63%) were both literate and mobile phone users. Of these, 91% (527/ 576) responded favourably to the potential use of SMS for health communication, while only 38.9% (124/319) of others were favourable to the idea (*p*<0.001). A lower proportion of literate mobile phone users reported optimal adherence to ART (86.4% vs. 90.6%; *p*=0.007). Male participants (AOR=2.81; 95% CI 1.83–4.30), sub-optimal adherence (AOR=1.76; 95% CI 1.12–2.77), those with waged or salaried employment (AOR=2.35; 95% CI 1.23–4.49), crafts/trade work (AOR=2.38; 95% CI 1.11–5.12), or involved in petty trade (AOR=1.85; 95% CI 1.09–3.13) (in comparison to those with no income) were more likely to report mobile phone ownership and literacy.

**Conclusions:**

In a rural Ugandan setting, we found that over 60% of patients could potentially benefit from a mobile phone-based ART adherence support. However, support for such an intervention was lower for other patients.

## Introduction

Despite the dramatic expansion of access to antiretroviral therapy (ART) in recent years, HIV/AIDS continues to be a major global health problem, especially in sub-Saharan Africa. Non-adherence to ART has been identified as a key barrier to successful treatment of HIV, which can lead to the development of virologic resistance and treatment failure [1]. Non-adherence can also increase the risk of onward transmission of HIV. Effective and efficient methods to improve adherence are needed in order to maximize the benefits of ART. Several methods have been proposed and implemented to support ART adherence in resource-limited settings by healthcare providers, including directly observed therapy, treatment supporters (providing different forms of support such as psychosocial support or HIV/ART education), and availability of education and counselling [2]. Methods requiring less intensive resources include “medicine companions” [3], reminder devices [4], HIV-specialized pharmacies [5] and automated voice messaging [6], among others.

Recently, the use of short message service (SMS) to send supportive messages through mobile phones have been examined for their potential use to improve adherence to ART; several meta-analyses have found weekly SMS messaging to be efficacious in promoting ART adherence [7–9]. A randomized trial in Kenya found that those who received weekly SMS communications with healthcare worker follow-up as needed performed significantly better in terms of ART adherence and virologic suppression in comparison to participants randomized to the control arm [10]. Another study in rural Kenya found increased adherence rates with the use of a similar intervention [11]. However, other studies using various forms of SMS communications have not been able to demonstrate similar levels of effectiveness [12,13]. Success of such initiatives depends on the various needs of the patients and the degree of support provided, which can be difficult for a weekly SMS message to incorporate; the difference in the results found by these studies have not been elucidated clearly due to these considerations. Some social factors found to be associated with the benefits of SMS on ART adherence include sex, level of education and timing/frequency of the SMS messaging [14].

The low cost and relative ease of sending SMS to mobile phones make it an attractive intervention for supporting ART adherence. Mobile phones are quickly becoming widespread in all areas of the world, with an estimated 7 billion mobile phone subscriptions as of 2014 [15]. While mobile phone use in Africa is lower than in other parts of the globe, still about 63% of the population is a mobile phone user [15]. This number is expected to continue to increase as most of Africa has bypassed the landline phase of telephones entirely [15]. The United Nations Joint Programme on HIV/AIDS (UNAIDS) and WHO have already included wireless communication technologies in strategic plans as a potential means of adherence and health promotion [16].

While these results seem promising in identifying an innovative and effective method of ART adherence, there are still many issues to address in terms of feasibility and generalizability. Although mobile phones are widely used in Africa, the penetrance into rural areas remains low; and many of those living in rural areas also demonstrate lower literacy rates than those in urban areas [17]. Acceptability to the patients does not seem to be a major issue, as previous studies investigating the use of SMS messaging for ART adherence found that patients viewed SMS messaging as helpful for increased adherence and appreciated it, although concerns about patient privacy were mentioned [18,19]. The studies examining the utility of mobile phones as a support to ART adherence undertaken to date have concentrated on dense, urban areas where mobile phone usage tends to be high. Our objectives in this study were to determine the proportion of people living with HIV who are literate and also use mobile phones in a rural setting in Uganda. This information will indicate the number of people that can be reached and helped with adherence issues with interventions involving SMS. We were primarily interested in the proportion and characteristics of HIV patients who could potentially benefit from SMS reminders, and whether such an intervention would be acceptable to this patient population.

## Methods

### Study setting

All subjects surveyed were receiving ART from The AIDS Support Organization (TASO) in Jinja, Uganda. TASO is the oldest and largest non-governmental HIV care and treatment organization in Uganda and provides treatment and support to over 100,000 HIV patients through 11 service centres across the country. The ART programme at TASO-Jinja began in 2004. Clients are provided with ART after meeting eligibility criteria as defined by the Ugandan Ministry of Health which currently restrict ART use to individuals with WHO stage III or IV illness or a CD4 cell count ≤350 cells/µL. Clients receiving ART are followed at visits of one to three months to refill medications and for clinical monitoring. CD4 cell counts are measured every six months and no routine viral load (VL) monitoring is provided. Jinja is a moderate-sized town (population 90,000) in the eastern region of the country which is approximately 80 km from the capital, Kampala.

### Participants

Beginning in June 2012 all clients of TASO-Jinja who had been receiving ART for four years, or more, were offered the opportunity to enrol in the Long Term Outcomes of ART in Uganda Study (LTOS), a three-year observational cohort study to identify the frequency of virologic failure in those receiving first-line ART and the determinants of treatment success for those starting on second-line treatment. After providing informed written consent in one of three local languages, participants were enrolled in this study from June 2012 to August 2013.

### Ethics approval

The study received approval from the Research Ethics Board of the University of British Columbia in Vancouver, Canada, and the Science and Ethics Committee of the Uganda Virus Research Institute and the Uganda National Council for Science and Technology in Uganda.

### Data collection

Using data collected at the first study visit, we measured self-described adherence behaviour, current adherence aids and socio-demographic characteristics of study participants using an interviewer-administered questionnaire and conducted a VL test. Optimal adherence was defined as missing an ART dose at most once a month. Participants were asked a multiple-choice question about how often they missed ART, with four choices: never, once a month or less, more than once a month but less than once a week, or once a week or more. The primary outcome measure was defined of a composite of whether participants had a mobile phone and whether they could read, as these would be individuals who could potentially benefit from a mobile phone text message reminder system. Participants were asked at the initial screening visit “Do you have a mobile phone?”, “Are you able to read?” and “Are you able to write?,” and we derived cell phone ownership and literacy (defined as being able to read and write) data from the answers. We also asked participants whether they would like TASO to use mobile phones for adherence support, with the question phrased as “Some ART programs have found it useful to use mobile phones to remind clients to take their ARTs or to check their health. Would you like TASO to use mobile phones for this purpose?” We measured HIV VL and CD4 cell counts and conducted clinical assessments at the time of entry into the study.

### Data analysis

We performed descriptive statistics using proportions for categorical variables and medians and interquartile ranges (IQR) for continuous variables. We conducted bivariate analyses using Pearson's chi-square and Fisher's exact tests for categorical variables, and the Wilcoxon rank-sum test for continuous variables to compare study participants who had a mobile phone and could read and write, with those who did not have a mobile phone and/or were not literate. We then conducted a logistic regression analysis to look for independent associations with the primary outcome. All variables found to be associated with the outcome at the *p*<0.10 level in the univariate analysis were considered for inclusion in the multivariate model, with the final model chosen using the minimization of Aikake Information Criterion. Statistical significance was set at a *p*≤0.05 for the multivariate model. All statistical analyses were carried out using SAS version 9.3 (SAS Institute; Cary, North Carolina, USA).

## Results

We enrolled 895 participants, of whom 684 (76.4%) were female. The median age was 44 years (IQR 44–50 years). Participants had been receiving ART for a median of 6.8 years (IQR 5.8–7.7 years) at study enrolment. Self-reported adherence to ART was generally very good with 776 (86.7%) of patients reporting missing a pill at most once a month. Overall, 741 (82.8%) of study participants reported having a mobile phone and 653 (73.0%) of patients self-reported as being able to read and write. A total of 576 (64.4%) patients were both literate and mobile phone owners.

Table 1 shows the bivariate analysis comparing the 576 study participants who were literate and had mobile phones with the 319 who were either illiterate, did not have a mobile phone or both. We observed no differences in the median age between literate mobile phone users (44 vs. 44, *p*=0.295) and other study participants. The duration on ART was similar in both groups, with a median of 6.75 years for literate users and 6.83 years for others (*p*=0.105). A majority of literate cell phone users were favourable to the idea of SMS communication for reminders or to check health status (527 of 576, 91.7%), while 38.9% of all others preferred the idea (124/319) (*p*<0.001). Literate mobile phone users tended to have a higher level of education: 99.7% reported some form of education, whereas 58.3% of other participants reported no education whatsoever (*p*<0.001). There were statistically significant differences in the major sources of income between the two groups (*p*=0.005), with a smaller proportion of literate phone users (29.9%) deriving most of their income from agriculture in comparison to the other group (32.3%) and a larger proportion of literate phone users derived their incomes from salaried employment or trade work (13.5% and 7.5% vs.7.5% and 4.4%, respectively).

**Table 1:** Descriptive statistics comparing study participants who were literate and owned a mobile phone (Group 2) vs. all others (Group 1)

	Total		By group		
		
			Group 1 (%)	Group 2 (%)	
	*n*=895	%	*n*=319	*n*=575	*p*
Support for TASO's use of phones					
Yes	651	72.8	38.9	91.7	<0.001
Sex					
Female	681	76.2	88.4	69.3	<0.001
Education					
No education	188	21.0	58.3	0.3	<0.001
Some primary	393	44.0	34.8	49.0	
Some secondary	254	28.4	5.6	41.0	
Some post-secondary or above	60	6.7	1.3	9.7	
Primary source of income					
Agriculture and farming	275	30.8	32.3	29.9	0.005
Wage or salaried employment	102	11.4	7.5	13.5	
Crafts and trade work	57	6.4	4.4	7.5	
Petty trade – kiosks, stalls	227	25.4	23.8	26.2	
None	85	9.5	11.9	8.2	
Other	149	16.7	20.1	14.8	
Marital status					
Single	31	3.5	1.3	4.7	<0.001
Legally married	165	18.4	11.9	22.0	
Co-habiting	206	23.0	19.1	25.2	
Separated	139	15.5	18.8	13.7	
Divorced	2	0.2	0.3	0.2	
Widowed	352	39.3	48.6	34.2	
Good adherence to ARV					
Yes	774	86.4	90.6	84.2	0.008
First viral load test at enrolment					
<1000 copies	817	92.2	94.3	91.0	0.089
Opinion of ARV reminders					
Useful	446	49.8	36.7	57.1	<0.001
Most helpful in adherence					
Use of reminders	281	31.4	22.3	36.5	<0.001
TASO counsellor support	1	0.1	0.3	0	
Medicine companion support	79	8.8	9.1	8.7	
After brushing teeth	8	0.9	0.6	1.0	
Morning/evening prayer	39	4.4	3.1	5.0	
Personal Adherence Plan (HPP)	487	54.4	64.6	48.8	
Use of medical companion					
Yes	480	53.6	55.2	52.8	0.529

A smaller proportion of literate phone owners reported optimal adherence as defined above: 84.2% of literate phone users reported good ART adherence, while 90.6% of the others did (*p*=0.007). Similarly, a higher proportion of literate phone users had VL measurements of ≥1000 copies/mL (9.0%), compared to other study participants (5.7%). However, this difference was not statistically significant (*p*=0.089). When asked about which types of interventions were most useful in supporting adherence to ART, literate phone users found reminders in one form or another (e.g. notes, reminders from family) more useful (57.1% of literate phone users compared to 36.7% of all others, *p*<0.001). A larger proportion of literate phone users cited the use of reminders as the most important factor influencing their adherence to drugs (36.5% vs. 22.3% for others, *p*<0.001), above other methods such as medicine companions, with prayer, etc. Similar proportions of the two groups made use of a “medicine companion” as adherence support, with no significant difference being detected (44.8% of literate phone users using a companion, compared to 47.2% of others; *p*=0.529).

In the adjusted logistic regression model shown in Table 2, we found that men were more likely to be literate phone users (adjusted odds ratio [AOR]=2.81; 95% confidence interval [CI] 1.83–4.30) compared to women. We also found that those in waged/salaried employment (AOR=2.35; 95% CI 1.23–4.49), crafts/trade work (AOR=2.38; 95% CI 1.11–5.12) and petty trade (AOR=1.85; 95% CI 1.09–3.13) were more likely to be literate phone users in comparison to those with no income. Married participants tended to be more likely to be literate phone users when compared to single participants, but this association only approached statistical significance (AOR=1.44, *p*=0.088). Participants who were literate and had access to a mobile phone were also more likely to report sub-optimal drug adherence (AOR=1.76; 95% CI 1.12–2.77), as defined previously. The VL results at enrolment were not independently associated with the outcome in the final logistic model.

**Table 2:** Logistic regression analysis of factors associated with participants who are both literate and own a mobile phone

	Unadjusted odds ratio		Adjusted odds ratio	
		
	(95% CI)	*p*	(95% CI)	*p*
Sex				
Female	Reference		Reference	
Male	3.37 (2.29–4.96)	<0.001	2.81 (1.83–4.30)	<0.001
Primary source of income				
None	Reference		Reference	
Agriculture and farming	1.38 (0.84–2.26)	0.205	1.23 (0.73–2.06)	0.431
Wage or salaried employment	2.62 (1.40–4.91)	0.003	2.35 (1.23–4.49)	0.010
Crafts and trade work	2.54 (1.21–5.32)	0.014	2.38 (1.11–5.12)	0.026
Petty trade – kiosks, stalls	1.64 (0.98–2.74)	0.058	1.85 (1.09–3.13)	0.023
Other	1.08 (0.63–1.86)	0.769	1.13 (0.65–1.98)	0.661
Marital status				
Single/separated/divorced	Reference		Reference	
Legally married/co-habiting	1.71 (1.16–2.51)	0.007	1.44 (0.95–2.17)	0.088
Widows	0.79 (0.54–1.15)	0.209	0.94 (0.64–1.39)	0.762
Good adherence to ARV				
Yes	Reference		Reference	
No	1.75 (1.13–2.72)	0.012	1.76 (1.12–2.77)	0.015
First viral load test at enrolment				
<1000 copies	Reference			
≥1000 copies	1.64 (0.94–2.86)	0.081		

## Discussion

This study conducted in a rural Ugandan setting, found that a majority of participants (64%) surveyed were both literate and phone users, suggesting that the use of mobile phones to support adherence to ART is likely feasible even in non-urban settings in sub-Saharan Africa. Furthermore, patients who were literate and had mobile phones were more receptive to the use of SMS communications for adherence support and health status checks in this study. However, there remains a fairly substantial proportion (35.6%) of participants in our study who could not read and/or did not have access to a mobile phone in this setting, suggesting that additional means of supporting adherence among such individuals is needed. While it is possible that one might not need to be completely literate to use text messaging, support for this type of intervention was much lower (38.9%) in the latter group than the group who owned mobile phones and were literate (91.7%). The RCT in Kenya extended the reach of the intervention by allowing those who were illiterate or without phones to share phone access [10], which could be incorporated into future interventions in settings such as our study.

Interestingly we found that the literate phone users reported a lower adherence to ART, had a more favourable perception of the utility of reminders to support adherence to treatment and tended to have a lower proportion of virologic suppression. A possible explanation for these finding could be attributed to the occupations and lifestyles of the literate mobile phone users as they were found to be more involved in commerce and trade, which could result in a busier, more unstable lifestyle which may lead to missing medication.

Mobile phone usage found in our study (83% of study participants) was much higher than the most recently available data on mobile phone usage in Uganda of 53% [20] as of June 2014, suggesting that either mobile phone use is continuing to rapidly expand or TASO clients are more likely to use mobile phones than the general population. Mobile phone coverage in Uganda has developed extremely fast, with almost 100% of the population covered by mobile cellular networks, having increased from 20% in 2000 [21]; a lack of infrastructure is not foreseen to be a significant problem in communicating via SMS. Approximately 73% of adult Ugandans are literate (able to read and write) according to 2012 data [22], which is identical to the proportion we found in our study.

This study also helps elucidate the adherence support interventions which were reported to be the most beneficial in terms of supporting the long-term adherence to therapy and low levels of virologic failure that we regularly observed at TASO. Most patients said that a personal TASO counsellor was not helpful in keeping to their regimen, but about half of study participants still made use of a medicine companion as a reminder, despite being on ART for more than five years.

Our results are amongst the first on mobile phone usage to be reported from a rural setting and suggest that mobile phone-based adherence supports may be as feasible in these settings. Findings from this study can be used as an indicator for the feasibility of studies such as those carried out in Kenya [10,11]. Similar receptiveness to the use of mobile phone reminders have been found in urban populations of countries throughout sub-Saharan Africa such as in South Africa [23], further indicating the wide potential of mobile phones as an accepted health intervention method. The uses of SMS in HIV treatment support are diverse, ranging from ART reminders to communication of lab results [24]. The feasibility of simple mobile phone reminders have already been called into question, as a recent RCT in India found no evidence of improved adherence or health outcome with a weekly voice reminder and picture message [13]. The divergent results from the two studies in Kenya and India stem from the type of information communicated via SMS; while the Kenyan WelTel study used SMS for health status communication, the Indian study sought to actively check whether the patients had taken ART. Both studies sent SMS messages weekly, indicating that frequency of messaging likely did not play a major role. This study specifically asked participants about their receptiveness of SMS use for both ART reminders and health status communication, and considering the findings from India and the limited effectiveness of mobile phone messaging in HIV care [25], health communication may serve as a better alternative for a mobile phone-based intervention. This is further corroborated by the Kenyan WelTel study. However, the ART programme in Jinja has already been demonstrated to have excellent client retention (67% after five years of treatment) and very low rates of virologic failure (7% of clients with VLs≥1000 copies/mL after five years) [26]. It remains to be seen whether implementing a text messaging system for client support could improve on these already impressive results.

This study has several limitations. First, as this study was conducted amongst long-term participants in the ART programme in Jinja, they may not be completely representative of all ART clients who initiate therapy in the region. However, we do not expect that this would bias our results towards an increased prevalence of mobile phone owner and literacy, as the literate phone user group tended to have lower self-reported adherence than other study participants. Second, it is difficult to know how representative the participants in the TASO Jinja programme are in comparison to other HIV patients in Africa who reside in rural areas. The TASO centre is located in a moderate-sized town in eastern Uganda (population 90,000, in 2011) [27]. However, a majority of the participants using TASO services (60%) reside outside of Jinja town, in areas which are considered more rural. It is important to consider that all data collected in this study were self-reported, in the form of answers to a questionnaire. It is also unclear whether the participants would have preferred SMS for ART reminders, health status checks or both; the wording of the question did not discern between these two roles and answers were strictly limited to either “yes” or “no,” leaving unclear whether patients were receptive to SMS being used for these roles. Finally, it was noted in previous studies that developing countries in Africa have non-negligible levels of shared mobile phone ownership [10,28]. Unfortunately, the questionnaire used in this study did not ask participants regarding shared usage, only about ownership.

## Conclusions

In summary, our study found that a sizeable proportion of HIV/AIDS patients receiving ART could benefit from the use of mobile phone-based support even in a rural Ugandan setting. However, it is also important to recognize that a significant minority of individuals in this programme were not supportive of such support, and/or did not have access to mobile phones. The community-focused supports at TASO Jinja, which have resulted in very high proportions of client retention, low mortality and good virologic outcomes, provide additional examples of programme components which may foster good client outcomes among ART programme participants in such settings. Other methods of supporting adherence to ART, whether they are innovative technology or conventional methods, must be considered for these groups in combination with mobile communications for those who are capable of using them. For example, the WelTel SMS service in Kenya allows for shared phone access to extend the service beyond those with their own phones or those who may be limited by illiteracy. Nonetheless, the results from this study serve as promising indicators for the potential of mobile phone technology for HIV/AIDS care. From this, further research into the scalability of mobile phone communications – support for rural HIV patients in sub-Saharan Africa and the kind of support needed by the patients – is needed.
